# A Preliminary Report on the Combined Effect of Intra-Articular Platelet-Rich Plasma Injections and Photobiomodulation in Canine Osteoarthritis

**DOI:** 10.3390/ani13203247

**Published:** 2023-10-18

**Authors:** J. C. Alves, Ana Santos, L. Miguel Carreira

**Affiliations:** 1 Divisão de Medicina Veterinária, Guarda Nacional Republicana (GNR), Rua Presidente Arriaga, 9, 1200-771 Lisbon, Portugal; 2 Faculty of Veterinary Medicine, Lusófona University, 1749-024 Lisbon, Portugal; 3 Centro de Ciência Animal e Veterinária, Lusófona University, 1749-024 Lisbon, Portugal; 4 MED—Mediterranean Institute for Agriculture, Environment and Development, Instituto de Investigação e Formação Avançada, Universidade de Évora, Pólo da Mitra, Ap. 94, 7006-554 Évora, Portugal; 5 Faculty of Veterinary Medicine, University of Lisbon (FMV/ULisboa), 1300-477 Lisbon, Portugal; miguelcarreira@fmv.ulisboa.pt; 6 Interdisciplinary Centre for Research in Animal Health (CIISA), University of Lisbon (FMV/ULisboa), 1649-004 Lisbon, Portugal; 7 Anjos of Assis Veterinary Medicine Centre (CMVAA), 2830-077 Barreiro, Portugal

**Keywords:** dog, osteoarthritis, chronic pain, orthopedics, platelet-rich plasma, regenerative therapy, photobiomodulation

## Abstract

**Simple Summary:**

Osteoarthritis is a very common joint disease in dogs, and clinicians usually favor a multimodal approach for the management of the disease. There has been a growing interest concerning platelet-rich plasma and photobiomodulation, alongside an increasing body of evidence supporting their use. Although there are studies reporting the effect of these treatments individually, there is still a lack of information on their combined use. We aimed to evaluate the effect of the intra-articular administration of platelet-rich plasma, photobiomodulation, and their combined use in dogs with bilateral hip osteoarthritis. Our results show that combining the two treatments leads to greater, longer-lasting clinical improvements.

**Abstract:**

Osteoarthritis (OA) is highly prevalent in the canine population. Due to the multiple dimensions of the disease, a multimodal approach is usually favored by clinicians. To evaluate the combined treatment with intra-articular platelet-rich plasma (PRP) and photobiomodulation in dogs with bilateral hip OA, thirty dogs were assigned to a PRP group (PRPG, n = 10), a photobiomodulation group (PBMTG, n = 10), or a combined therapies group (PRP+PBMTG, n = 10). The PRPG received two intra-articular administrations of platelet-rich plasma 14 days apart. The PBMTG received photobiomodulation with a therapeutic laser, with three sessions every other day in week one; two sessions in week two; a single session in week three; and one session/month on follow-up evaluation days. The PRP+PBMTG received the two combined therapies. The response to treatment was evaluated with weight-bearing distribution and the Canine Brief Pain Inventory, the Liverpool Osteoarthritis in Dogs, and the Canine Orthopedic Index. Evaluations were conducted before treatment and +8, +15, +30, +60, and +90 days after initial treatment. Normality was assessed with a Shapiro–Wilk test, and the groups’ results in each evaluation moment were compared using a Mann–Whitney U test. Animals of both sexes (male n = 19, female n = 11) were included in the sample, with a mean age of 7.8 ± 2.5 years and a body weight of 26.5 ± 4.7 kg. Joints were classified as mild (n = 6, three in PRPG, two in PBMTG, and one in PRP+PBMTG), moderate (n = 18, six in PRPG, five in PBMTG, and seven in PRP+PBMTG), and severe (n = 6, one in PRPG, three in PBMTG, and two in PRP+PBMTG). No differences were found between groups at the initial evaluation. All treatments produced clinically significant improvements compared to the assessment on treatment day. The combination of PRP and photobiomodulation produced greater, longer-lasting improvements. PRP and photobiomodulation can improve objective outcomes and client-reported outcome measures in dogs with OA. Their combined use leads to greater, longer-lasting, clinically significant improvements.

## 1. Introduction

Osteoarthritis (OA) has a high prevalence in the canine population, and the disease significantly impacts the patient’s overall quality of life, as it produces pain and affects joint function and mobility [1,2,3,4]. Adequate disease management is still challenging, as reflected in the broad number of therapeutic approaches described [5,6]. Due to the multiple dimensions of the disease, a multimodal approach is usually favored by clinicians [7].

Autologous platelet therapies are an interesting approach, as platelets are a part of the body’s natural response to injury. Attributed effects include a reduction in inflammation and a contribution to tissue regeneration. At the joint level, platelet-rich plasma (PRP) can promote cartilage synthesis or inhibit its breakdown [8,9]. The use of PRP has been described for the treatment of different musculoskeletal conditions in dogs, such as OA, tendinopathies, or muscle injury [10,11], but the described effects vary significantly between reports. This variability is likely related to various compositions and characteristics of PRP products regarding concentration and numbers of platelet, leukocytes, and red blood cells [12,13].

Photobiomodulation therapy (PBMT) has also gained increasing interest based on the ability of red/near infrared light to produce a clinical effect, including stimulation of tissue healing, analgesia, and reduced inflammation [14]. It has been described in dogs as managing various conditions, such as OA, gingivostomatitis, wound healing, and even diarrhea [15,16,17,18,19]. The results obtained with PBMT in managing osteoarthritis are attributed to the effect of the delivered photons that dissociate inhibitory nitric oxide while increasing electron transport and ATP production. It also increases the expression of genes that increase protein synthesis, particularly anti-apoptotic proteins, and antioxidant enzymes, leading to cell proliferation and anti-inflammatory signaling [15]. As for PRP, a variability in described effects of PBMT is also found in the available literature, likely linked to the difference in selected parameters [17].

Having objective measures to evaluate patients and determine response to treatment is paramount. The evaluation of weight bearing, off-loading, and limb favoring are evaluations commonly performed during the orthopedic exam [20,21]. In OA cases, some patients may exhibit only discrete lameness at a walk or a trot while showing changes in weight-bearing distribution at a stance in response to pain [22,23]. In fact, stance analysis has been shown to be sensitive in identifying dog lameness [24]. In addition to objective measures, several client-reported outcome measures have been developed to assess OA patients. They can identify changes and degrees of a pet’s subjective status, and owners can also interpret changes over an extended period of time [25,26]. The client-reported outcome measures aimed at dogs with OA include the Liverpool Osteoarthritis in Dogs (LOAD) [27,28], the Canine Orthopedic Index (COI) [29], and the Canine Brief Pain Inventory (CBPI) [30]. They have been recommended for use in dogs with OA in a recent COSMIN-based systemic review [31] and in WSAVA guidelines for the recognition assessment and treatment of pain [32].

This study aimed to evaluate the combined treatment with intra-articular PRP and PBMT in dogs with bilateral hip OA. It will serve as a preliminary study to determine adequate treatment parameters and frequency. We hypothesized that combining the two treatments would better alleviate OA-related clinical signs than their isolated use.

## 2. Materials and Methods

The study protocol was approved by the ethical review committee of the University of Évora (Órgão Responsável pelo Bem-Estar dos Animais da Universidade de Évora, approval no. GD/16901/2022). All methods were carried out in accordance with relevant guidelines and regulations, complying with ARRIVE guidelines. Informed consent and permission were obtained in writing from the institution responsible for the animals (Guarda Nacional Republicana, Portuguese Gendarmerie).

Thirty dogs with bilateral hip OA were recruited. All animals had a consistent history, with the canine handler trainer referring specific complaints, such as difficulty rising, jumping, and maintaining obedience positions. Pain during joint mobilization, stiffness, and reduced range of motion was elicited on physical examination. Radiographic findings were consistent with bilateral hip OA (Orthopedic Foundation for Animals’ hip scores of mild, moderate, or severe) [33]. Animals were >2 years old, had a body weight > 20 kg, and were without any other medications or nutritional supplements administered for >6 weeks. Animals with other orthopedic, neurologic, or other diseases were excluded. Since all animals had clinical signs of OA, a placebo group was not included for ethical reasons. However, these treatments have been evaluated compared to a placebo or control before [11,16].

After selection, patients were randomly assigned a PRP group (PRPG, n = 10), a PBMT group (PBMTG, n = 10), or a combined therapies group (PRP+PBMTG, n = 10). Since all animals had bilateral hip OA, both joints of each patient received the same treatment [11,16]. The PRPG received two intra-articular administrations of 2 mL of PRP per hip joint, produced with the commercially available CRT PurePRP^®^ Kit (Companion Regenerative Therapies, Newark, DE, USA). One was administered on day 0, and, in accordance with the manufacturer’s recommendations, a follow-up administration was performed 14 days after the initial treatment. For the preparation of PRP, 50 mL of whole blood was collected from the patient’s jugular vein directly into a 60 mL syringe filled with 10 mL of Anticoagulant Citrate Dextrose Solution. After collection, the blood was transferred and loaded into a concentrating device. The device was then placed in a centrifuge (Executive Series Centrifuge II, Companion Regenerative Therapies, Newark, DE, USA) and spun at 3600 rpm for 1 min. After the first centrifugation, the buffy coat and platelet-poor plasm were collected, transferred to a second concentrating device, and spun at 3800 rpm for 5 min. After the second centrifugation process, the remaining platelet-poor plasma was removed until 4 mL was left. The device was then swirled to resuspend the platelets, and the 4 mL of PRP was aspirated into a 12 mL syringe. The PRP was administered immediately after preparation, without activation.

PRP and whole blood samples were sent to an external lab for analysis. Compositions were determined and compared. The intra-articular administration was conducted under light sedation, obtained with the simultaneous intravenous administration of medetomidine (0.01 mg/kg) and butorphanol (0.1 mg/kg). The procedure for hip intra-articular administrations has been described before [6]. The animal was placed in lateral recumbency, with the joint being assessed at that moment upward. A 4 × 4 cm window, with the greater trochanter in the center, was clipped and aseptically prepared. An assistant then placed the limb in a neutral position parallel to the table. A21-gauge with a 2.5″ length needle was then introduced just dorsal to the greater trochanter, perpendicular to the limb’s long axis until the joint was reached. Correct needle placement was confirmed by collecting synovial fluid.

The PBMTG received PBMT with a therapeutic laser (CTS-DUO Class IV Laser, Companion Animal Health, Enovis, Wilmington, DE, USA). The hair was clipped (for blinding purposes), and no sedation was required for PBMT. Sessions were conducted for three consecutive weeks in the following fashion: in week one, three sessions every other day; in week two, two sessions, two days apart; in week three, a single session. After this first treatment period, a single session was conducted monthly on follow-up evaluation days. PBMT parameters are presented in Table 1 and were selected based on the manufacturer’s recommendation and previous evidence of a positive therapeutic effect [16].

Animals in the PRP+PBMTG were treated with the two combined therapies on the same schedule as the PRPG and the PBMTG. All groups were prescribed a 3-day rest period following the days of the IA administrations for the PRPG and the PRP+PBMTG.

The follow-up evaluations were conducted at scheduled moments on days 14 (+14 d, before the second IA PRP administration), 30 (+30 d), and 90 (+90 d) after the initial treatment. At the follow-up moments, a weight-bearing evaluation was conducted (Companion Stance Analyser; Enovis^®^, Newark, DE, USA). The procedure of weight-bearing evaluation was performed as described before [34]. The equipment was placed in the center of a room, at least 1 m from the walls. After zeroing the equipment, dogs were encouraged to stand on the platform, ensuring that one foot was placed on each quadrant. At least twenty measurements were obtained for each patient, and the mean value was determined. After weight-bearing distribution values were collected, a deviation from the normal weight distribution for pelvic limbs was calculated by subtracting the weight-bearing of the limb from the considered normal of 20% [23]. In addition, a left-right symmetry index (SI) was also calculated with the formula: SI = [(WBR − WBL)/((WBR + WBL) × 0.5)] × 100, where WBR is the weight-bearing for the right limb, and WBL is the weight-bearing for the left limb [28,35]. Negative values were made positive. In addition to the weight-bearing distribution evaluation, a digital copy of the CBPI, the LOAD, and the COI were completed sequentially by the same handler, blinded to their dog’s treatment group. All have previously been validated in Portuguese versions [36,37,38,39].

All of the described procedures were performed by the same researcher, who was kept blinded to the dogs’ treatment group. Data were assessed with a Shapiro–Wilk test for the evaluation of normal distribution. In each evaluation moment, the groups’ results were compared using a Mann–Whitney U test. All results were analyzed with IBM SPSS Statistics version 20. Statistical significance was considered at *p* < 0.05.

## 3. Results

Animals of both sexes were included in the present sample (18 males, eight in PRPG, five in PBMTG, and five in PRP+PBMTG; and 12 females, two in PRPG, five in PBMTG, and five in PRP+PBMTG), having a mean age of 7.8 ± 2.5 years (7.6 ± 1.9 in PRPG, 7.9 ± 2.4 in PBMTG, and 7.4 ± 2.8 in PRP+PBMTG). All had an ideal body condition score for working dogs of 4/9 (n = 24) and 5/9 (n = 6) on the Laflamme scale [40], with a mean body weight of 26.5 ± 4.7kg (26.4 ± 2.4 in PRPG, 27.3 ± 5.2 in PBMTG, and 24.9 ± 4.6 in PRP+PBMTG). Four dog breeds were present in the sample, similar to those found in most police and working dog populations throughout the world: German Shepherd Dogs (n = 11, three in PRPG, four in PBMTG, and four in PRP+PBMTG), Belgian Malinois Shepherd Dogs (n = 9, four in PRPG, two in PBMTG, and three in PRP+PBMTG), Labrador Retriever (n = 6, two in PRPG, two in PBMTG, and two in PRP+PBMTG), and Dutch Shepherd Dogs (n = 4, one in PRPG, two in PBMTG, and one in PRP+PBMTG). The dogs were used in drug detection and patrol work. Hips were graded with the OFA hip grading scheme, and six animals were classified as mild (three in PRPG, two in PBMTG, and one in PRP+PBMTG), 18 as moderate (six in PRPG, five in PBMTG, and seven in PRP+PBMTG), andsix as severe (one in PRPG, three in PBMTG, and two in PRP+PBMTG). All patients were followed up to the last evaluation moment (+90 days), and during this period, no additional treatment or medications were administered.

The composition of whole blood and PRP is presented in Table 2. Preparation of PRP took around 20 min, from the initial blood collection to the final administration.

The results of the weight-bearing evaluation and scores of the different client-reported outcome measures in each group are presented in Table 3. No significant differences were observed on day 0. While all treatments produced clinically significant improvements compared to the evaluations on day 0, the combination of PRP and PBMT had greater and longer-lasting effects.

**Figure 1:**
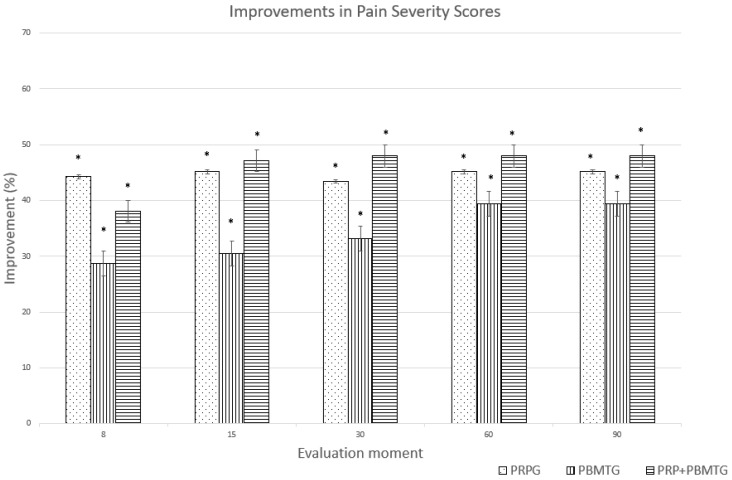
Improvements (%) in Pain Severity Score for the platelet-rich plasma group (PRPG), the photobiomodulation group (PBMTG), and the combined therapies group (PRP+PBMTG), compared to baseline values. * indicates a clinically significant improvement (reduction ≥ 1).

**Figure 2:**
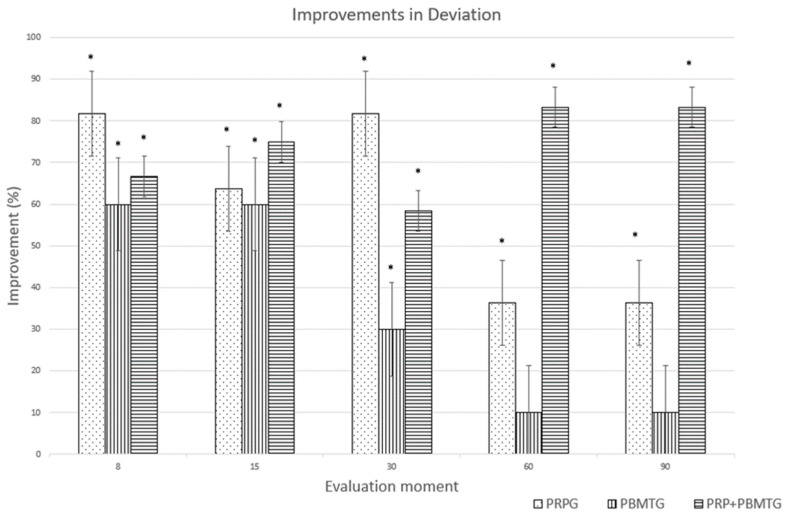
Improvements (%) in deviation in weight-bearing for the platelet-rich plasma group (PRPG), the photobiomodulation group (PBMTG), and the combined therapies group (PRP+PBMTG), compared to baseline values. * indicates a clinically significant improvement (improvement ≥ 1).

The improvements in PSS and LOAD for each group are presented in Figure 1 and Figure 2, respectively. With the CBPI, a reduction of ≥1 in PSS and ≥2 in PIS was considered a clinically-important change [41]. The same level has been determined for the LOAD and COI, suggested as a reduction of ≥4 and ≥14, respectively [42]. Unreported data from our group indicate that clinically important changes consist of improvements of ≥1 for deviation and ≥10 for SI in dogs with OA.

## 4. Discussion

Clinicians usually prefer multimodal approaches in managing OA to cover the multiple dimensions of the disease and to gather the benefits of each therapeutic approach, minimizing possible side effects. Our results show that the combined treatment with PRP and PBMT leads to greater and longer-lasting clinically significant improvements compared to their individual use.

Some reports are available on the individual use of these therapies in managing OA. Regarding intra-articular PRP, many are based on surgically induced models [43,44,45], but there are also reports available for dogs with naturally occurring OA. As a whole, these products have been able to improve clinical signs at the 12-week evaluation post-treatment or longer [11,46,47]. Our results show that PRP can produce a clinically significant improvement with objective outcome measures and client-reported outcome measures. These improvements lasted until the last evaluation moment, a result in line with the previous report showing a long-lasting therapeutic effect [11].

Similarly, PBMT has been shown to improve pain levels and lameness in dogs with OA alone or in conjunction with NSAID [15,16]. These beneficial effects were observed during treatment but tended to wean out after treatment was discontinued [16]. Our results show that a continued PBMT protocol can improve clinical signs and objective outcome parameters in dogs with hip OA, with long-lasting effects. In fact, pain severity scores showed a continuous improvement over time. It would be interesting to evaluate if this effect persists with time, and future studies should include a longer follow-up period to confirm this finding.

It was interesting to observe that the combined use of PRP and PBMT led to a sustained and consistently greater improvement. At a clinically significant level, this improvement was observed in both objective outcome measures and client-reported outcome measures. A previous report has shown that the concomitant use of PBMT with an NSAID has led to lower doses of NSAID being required to maintain an adequate response to treatment [15]. In our study, lower doses of medication do not apply. Still, improvements are observed with several outcome measures, in many cases from the first follow-up moment and lasting up to the +90 d evaluation. There are several references available reporting that PBMT is commonly employed in conjunction with other treatment modalities for the management of human and animal OA, including therapeutic exercises [48], oral joint supplements [49], and biological treatments [50] that show improved results with the combined therapies. These improvements result from increased downregulation of pro-inflammatory cytokines and metalloproteinases, upregulation of tissue inhibitors of metalloproteinases, and prevention of joint degeneration [50].

The reason for this synergetic effect is not completely clear. Suggested effects of PBMT include stimulation of tissue healing, analgesia, and reduced inflammation [14,51,52]. These effects are attributed to ATP, NO, and reactive oxygen species within cells, altering gene transcription, increasing cell proliferation, and producing growth factors [53]. Similarly, platelets may contribute to tissue regeneration, reduce local inflammation, and to the synthesis of cartilage or inhibition of its breakdown [54], effects mediated by growth factors [55,56]. It is possible that some of the greater improvements are obtained through an increased reduction in inflammation, obtained from the combined use of the two treatment modalities, which act in different pathways. They can also have combined action on inhibiting cartilage breakdown and increasing tissue healing. Another possibility is that PBMT may help improve the degranulation of the platelet’s alpha granules or provide a more favorable “field” for liberated growth factors to act. Future studies should include a longer follow-up period to determine if this improvement level is maintained in time.

Although an overall improvement has been observed with most outcome measures considered, improvement with SI and the LOAD seem less remarkable. Some reasons may account for this finding. It has been described that even dogs without OA show some level of asymmetry, up to a 10% level [57]. Although this value was observed with different equipment, a similar phenomenon may be observed with our results. Although it was not a significant difference, a variation in SI was observed between groups at 0 d, but not with deviation. It has been described that dogs with bilateral hip OA can exhibit different compensation mechanisms, even in cases with the same hip OA grade. Some can show side-to-side compensation, while others exhibit pelvic-to-thoracic compensation [34]. This emphasizes the importance of evaluating SI and deviation to obtain an individualized evaluation of each animal evaluation, rather than relying exclusively on expected compensations [41]. With the LOAD, values at the first assessment were relatively low, making it harder to observe a significant improvement. In fact, it has been suggested that including animals with higher client-reported outcome measures scores is preferred, as it increases the likelihood of detecting a clinically significant improvement [27].

We observed some side effects following the intra-articular administration, with some patients showing complaints that resolved without external intervention within 24–72 h. These side effects are similar to those reported following PRP administration, which includes injection pain and local inflammation. They are local, transient, and self-limiting, taking 2–10 days to resolve [58]. We did not record if these complaints were lower in the PRP+PBMT than the PRPG, but this should be evaluated in future studies. Similarly, a power analysis should also be included.

## 5. Conclusions

This study showed that PRP and PBMT could improve objective outcomes and client-reported outcome measures in dogs with OA. Their combined use leads to greater, longer-lasting, clinically significant improvements. Future studies should address the limitations pointed out in the presented study, including a longer follow-up period to evaluate the duration of the observed improvements in all considered groups and a calculation of power analysis and sample size.

## Figures and Tables

**Table 1:** Photobiomodulation therapy treatment parameters.

	Light Parameters (Dose)
Wavelength (nm)	980 nm
Radiant Power (W)	13
Irradiance (W/cm^2^) at the skin surface	2.6 (using a large contact treatment head)
Fluence (J/cm^2^)	15
Total Joules	5250
Treatment Protocol	Continuously moving grid pattern in contact over the treatment area at a speed of 2.5–7.5 cm/s, according to manufacturer recommendations.
Treatment Area (cm^2^)	350 (entire hip area)
Treatment Time	6 min, 44 s

**Table 2:** Mean values (±standard deviation) of whole blood and platelet-rich plasma product composition.

Parameter	Whole Blood		Platelet Concentrate	
	Mean Value	SD	Mean Value	SD
Platelets (×10^3^/mm^3^)	298.12	78.30	1553.21	400.90
RBC (×10^6^/mm^3^)	6.20	1.10	0.50	0.06
WBC (×10^3^/mm^3^)	10.16	3.96	4.07	3.09
Lymphocytes (×10^3^/mm^3^)	2.05	0.78	2.46	1.63
Monocytes (×10^3^/mm^3^)	0.69	0.37	0.52	0.31
Neutrophils (×10^3^/mm^3^)	7.03	3.17	0.77	0.32
Eosinophils (×10^3^/mm^3^)	0.38	0.43	0.32	0.37
Basophils (×10^3^/mm^3^)	0.01	0.02	0.00	0.00

**Table 3:** Evolution of weight-bearing results and the considered client-reported outcome measures (median, inter-quartile range, and percentual change) by group and moment. CBPI—Canine Brief Pain Inventory; COI—Canine Orthopedic Index; LOAD—Liverpool Osteoarthritis in Dogs; PIS—Pain Interference Score; PSS—Pain Severity Score; QOL—Quality of Life. * indicates significance when comparing groups at each follow-up moment.

Measure		Group	T0		*p*	+8 d			*p*	+14 d			*p*	+30 d			*p*	+60 d			*p*	+90 d			*p*
			Med	IQR		Med	IQR	%		Med	IQR	%		Med	IQR	%		Med	IQR	%		Med	IQR	%	
Weight-bearing	Symmetry Index	PRPG	19.8	40.3	0.85	5.1	5.3	74.1	0.01 *	9.5	5.3	51.8	0.04 *	5.4	13.4	72.6	0.04 *	10.3	24.5	48.2	0.04 *	10.3	24.5	48.2	0.04 *
		PBMTG	20.3	25.3		7.2	13.3	64.5		5.1	5.4	74.7		8.8	12.6	56.6		9.1	12.5	55.1		9.1	12.5	55.1	
		PRP+PBMTG	29.3	26.7		5.3	5.3	82.0		5.0	7.2	82.9		5.3	8.1	82.0		5.1	13.2	82.5		5.1	13.2	82.5	
	Deviation	PRPG	5.5	2.8	0.08	1.0	1.0	81.8	0.24	2.0	1.8	63.6	0.74	1.0	2.3	81.8	0.03 *	3.5	5.5	36.4	0.01 *	3.5	5.5	36.4	0.01 *
		PBMTG	5.0	2.8		2.0	2.8	60.0		2.0	1.0	60.0		3.5	4.5	30.0		4.5	3.8	10.0		4.5	3.8	10.0	
		PRP+PBMTG	6.0	4.5		2.0	1.5	66.7		1.5	1.0	75.0		2.5	4.3	58.3		1.0	3.8	83.3		1.0	3.8	83.3	
CBPI	PSS (0–10)	PRPG	5.3	0.7	0.37	3.0	0.4	44.3	0.71	2.9	0.6	45.1	0.63	3.0	0.8	43.4	0.80	2.9	1.1	45.1	0.76	2.9	1.1	45.1	0.76
		PBMTG	5.1	1.7		3.6	1.7	28.7		3.5	1.7	30.5		3.4	1.9	33.2		3.1	1.8	39.4		3.1	1.8	39.4	
		PRP+PBMTG	5.5	1.0		3.4	0.7	38.0		2.9	1.5	47.1		2.9	1.1	47.9		2.9	1.1	47.9		2.9	1.1	47.9	
	PIS (0–10)	PRPG	4.0	1.0	0.08	1.3	1.3	68.8	0.04 *	1.5	2.3	62.5	0.04 *	1.5	2.4	62.5	0.03 *	2.3	1.8	43.8	0.03 *	2.3	3.3	43.8	0.03 *
		PBMTG	4.5	2.3		2.6	4.8	41.7		4.4	4.8	2.8		2.9	4.1	36.1		2.9	3.6	36.1		2.9	3.6	36.1	
		PRP+PBMTG	5.0	1.7		1.1	2.3	77.5		1.0	4.9	80.0		1.0	3.4	80.0		1.0	1.1	80.0		1.0	1.1	80.0	
LOAD (0–52)		PRPG	20.0	7.5	0.06	13.0	2.0	35.0	0.04 *	13.5	5.3	32.5	0.04*	13.0	9.5	35.0	0.71	15.5	13.5	22.5	0.81	15.5	13.5	22.5	0.01 *
		PBMTG	23.5	12.0		16.5	15.3	29.8		17.5	16.8	25.5		16.5	15.5	29.8		13.0	14.3	44.7		13.0	14.3	44.7	
		PRP+PBMTG	26.5	7.0		12.0	3.5	54.7		12.0	15.3	54.7		12.0	10.0	54.7		11.5	11.5	56.6		11.5	11.5	56.6	
COI	Stiffness (0–16)	PRPG	3.2	1.3	0.47	1.5	2.0	53.1	0.04 *	1.4	3.7	56.3	0.04 *	1.5	2.5	53.1	0.03 *	2.6	2.6	18.8	0.03 *	2.6	2.6	18.8	0.03 *
		PBMTG	4.5	2.2		3.0	4.4	33.3		4.3	4.2	4.4		2.6	4.1	42.2		2.7	3.8	40.0		2.7	3.8	40.0	
		PRP+PBMTG	5.0	1.3		1.4	2.7	72.0		1.0	6.0	80.0		1.0	3.3	80.0		1.0	2.0	80.0		1.0	2.0	80.0	
	Function (0–16)	PRPG	7.0	3.8	0.20	4.0	3.8	42.9	0.03 *	4.0	4.8	42.9	0.03 *	4.0	4.0	42.9	0.04 *	4.0	5.3	42.9	0.82	4.0	5.3	42.9	0.82
		PBMTG	6.0	3.5		4.5	5.5	25.0		4.5	16.8	25.0		5.5	5.5	8.3		4.5	6.3	25.0		4.5	6.3	25.0	
		PRP+PBMTG	8.0	0.8		2.5	3.0	68.8		2.0	15.3	75.0		3.5	3.5	56.3		3.0	6.0	62.5		3.0	6.0	62.5	
	Gait (0–20)	PRPG	7.0	3.5	0.54	2.0	2.8	71.4	0.02 *	3.5	3.0	50.0	0.03 *	3.0	2.5	57.1	0.78	4.0	6.0	42.9	0.71	4.0	6.0	42.9	0.02 *
		PBMTG	6.0	3.8		4.0	6.8	33.3		4.5	6.8	25.0		4.5	6.0	25.0		5.5	6.0	8.3		5.5	6.0	8.3	
		PRP+PBMTG	7.5	1.8		1.0	3.0	86.7		2.5	5.5	66.7		3.5	4.0	53.3		3.5	4.0	53.3		3.5	4.0	53.3	
	QOL (0–12)	PRPG	7.0	2.0	0.71	4.5	3.5	35.7	0.04 *	4.5	7.0	35.7	0.02 *	4.5	4.8	35.7	0.01 *	7.5	8.0	−7.1	0.03 *	7.5	8.0	−7.1	0.01 *
		PBMTG	8.0	5.3		5.5	6.5	31.3		6.0	5.8	25.0		5.0	5.8	37.5		7.0	5.8	12.5		7.0	5.8	12.5	
		PRP+PBMTG	8.0	2.3		2.5	4.5	68.8		2.5	10.0	68.8		3.5	4.0	56.3		3.5	6.3	56.3		3.5	6.3	56.3	
	Overall (0–64)	PRPG	41.0	4.2	0.23	23.5	3.0	42.7	0.03 *	25.5	5.0	37.8	0.01 *	24.5	5.2	40.2	0.01 *	31.0	8.2	24.4	0.03 *	31.0	8.2	24.4	0.04 *
		PBMTG	43.5	6.1		30.5	8.5	29.9		45.5	11.5	−4.6		31.5	8.2	27.6		30.0	8.1	31.0		30.0	8.1	31.0	
		PRP+PBMTG	50.0	2.9		18.0	3.5	64.0		29.0	11.5	42.0		22.5	5.4	55.0		21.5	6.9	57.0		21.5	6.9	57.0	

## Data Availability

All data generated or analyzed during this study are included in this published article.
